# Aneurysmal bone cyst of the rib. Robotic resection of a rare lesion

**DOI:** 10.1093/jscr/rjaf514

**Published:** 2025-07-14

**Authors:** Luis Arana-Bolaños, Xcaret Luna-Vargas, Amelia Fernández-Avendaño, Mónica Martínez-Ferman, Pablo Gomes-da Silva de Rosenzweig, Francina Bolaños-Morales

**Affiliations:** Department of Thoracic Surgery, Instituto Nacional de Enfermedades Respiratorias Ismael Cosío Villegas, Calz. de Tlalpan 4502, Ciudad de México, Mexico; Department of Thoracic Surgery, Instituto Nacional de Enfermedades Respiratorias Ismael Cosío Villegas, Calz. de Tlalpan 4502, Ciudad de México, Mexico; Department of Thoracic Surgery, Instituto Nacional de Enfermedades Respiratorias Ismael Cosío Villegas, Calz. de Tlalpan 4502, Ciudad de México, Mexico; Department of Thoracic Surgery, Instituto Nacional de Enfermedades Respiratorias Ismael Cosío Villegas, Calz. de Tlalpan 4502, Ciudad de México, Mexico; Department of Thoracic Surgery, Instituto Nacional de Enfermedades Respiratorias Ismael Cosío Villegas, Calz. de Tlalpan 4502, Ciudad de México, Mexico; Department of Thoracic Surgery, Instituto Nacional de Enfermedades Respiratorias Ismael Cosío Villegas, Calz. de Tlalpan 4502, Ciudad de México, Mexico

**Keywords:** aneurysmatic bone cyst, robotic surgery, bone tumor, primary thoracic tumor, minimally invasive surgery

## Abstract

Primary chest wall tumors are rare, with rib involvement accounting for 5%–7% of primary bone tumors. Aneurysmal bone cysts typically present as a rapidly growing, destructive mass that may infiltrate surrounding tissues. We present the case of a 21-year-old female with chronic pain in the left hemithorax caused by an aneurysmal bone cyst originating from the seventh rib arch. A minimally invasive en bloc resection was successfully performed using a robotic platform. Robot-assisted surgery offers a promising therapeutic approach for patients with well-defined chest wall tumors, allowing for a less invasive procedure and avoiding the need for more extensive techniques that may increase morbidity or necessitate chest wall reconstruction.

## Introduction

Primary chest wall tumors (PCWTs) encompass a diverse range of pathologies originating from soft tissue, vascular structures, bone, or cartilage (Table 1). These tumors account for only 0.04% of all newly diagnosed cancers [1], 5% of thoracic neoplasms, and 1%–2% of primary tumors [2]. Up to 60% of PCWTs are malignant [1], with more than half being metastatic lesions originating from distant organs or arising from local invasion by adjacent structures such as the breast, pleura, lung, or mediastinum [3].

**Table 1:** Chest wall tumors

	**Bone**	**Soft tissues**
**Benign**	Fibrous dysplasia Osteochondroma Chondroma Aneurysmal bone cyst	Lipoma Fibroma Hemangioma Giant cell tumor
**Malignant**	Chondrosarcoma Ewing sarcoma Osteosarcoma Solitary plasmacytoma	Fibrous histiocytoma Liposarcoma Fibrosarcoma

Aneurysmal bone cysts are a rare entity within primary bone tumors, accounting for 1.3% of all primary bone tumors. They predominantly affect long bones and vertebrae, with only 2.7% involving the ribs [4]. These cysts are characterized by blood-filled lesions lacking an endothelial cell lining, and exhibit a rapid and destructive growth with the potential to infiltrate adjacent tissues [5]. Due to the rarity of this pathology, no consensus exists regarding its management and treatment.

This article presents the case of a 21-year-old female with chronic pain, ultimately diagnosed with an aneurysmal bone cyst. The lesion was successfully resected using robotic-assisted surgery (RATS).

## Case report

**Figure 1:**
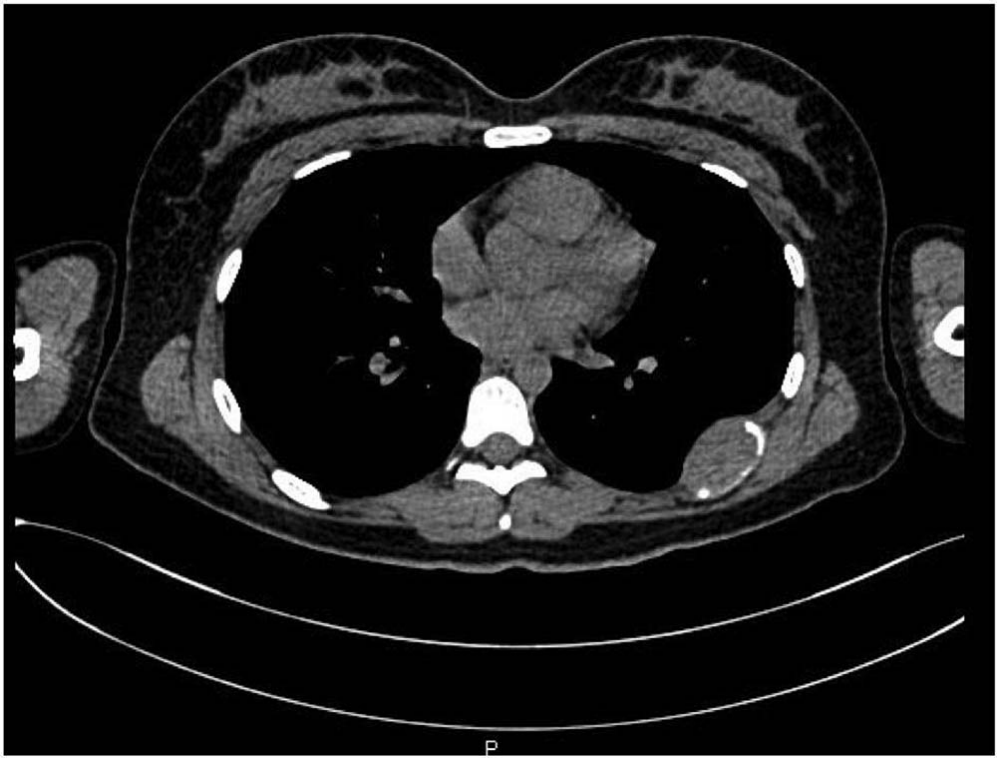
Transverse view of the lesion located on the seventh rib.

A 21-year-old female presented with a 5-month history of mild pain in the left hemithorax and paravertebral region. She was initially treated with nonsteroidal anti-inflammatory drugs without improvement. A chest X-ray (CXR) revealed a mass involving the seventh rib, leading to her referral to a tertiary care center. A computed tomography (CT) scan confirmed a tumor originating from the left seventh rib, measuring 21 mm × 38 mm (Fig. 1).

**Figure 2:**
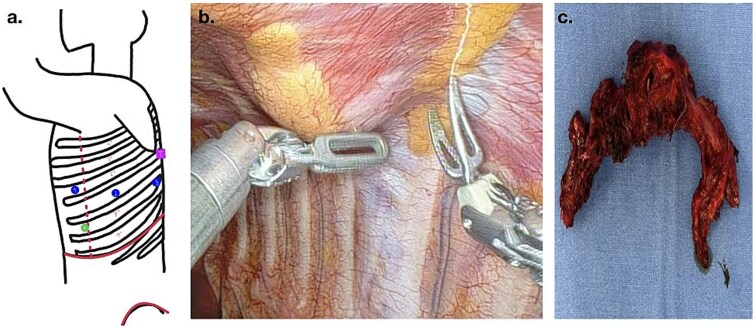
(a) Port placement for RATS access: the most inferior and anterior dot (green dot) indicates the camera port, the dots located at the middle of the scheme (blue) represent the robotic arm ports, and the most posterior and superior dot (pink) designates the area where the tumor was located; (b) robotic view of dissection; (c) surgical specimen extracted.

Surgical resection was planned due to suspected malignancy. The procedure was performed using the Da Vinci X robotic platform, employing three ports and an accessory device. The patient was positioned in a right lateral decubitus position, and key entry points to the thorax were marked (Fig. 2a and b). The tumor, measuring 5 cm × 4 cm, was located in the seventh rib (Fig. 2c). Dissection was performed, and the lesion was marked with endoclips. A laparoscopic rib cutter was used to excise the mass with a macroscopic margin of 2 cm. A 4 cm incision was made along the rib margin to exteriorize the lesion. Ports were closed using 3-0 Monocryl sutures up to the skin level. A 24FR Blake drain connected to a water seal was placed through the accessory port.

Pathological examination revealed a heterogeneous neoplasm featuring solid areas with multinucleated giant cells of the osteoclast-like type, cystic regions containing large, irregular vessels and extravasated red blood cells, reactive cortical bone at the periphery, and foci of mature hyaline cartilage. The diagnosis was consistent with an aneurysmal bone cyst.

Following the surgical procedure, the patient showed a favorable postoperative course. The pleural drain was removed on the second day, and she was discharged on the third postoperative day. The patient is continues to be in follow-up at our service.

## Discussion

PCWTs can be diagnosed at any age, with a predilection for specific age groups. In most cases, they are discovered incidentally, as they typically present as slow-growing, asymptomatic palpable masses [1, 2, 6]. However, they can cause pain due to growth and subsequent periosteal stretching or damage, regardless of their benign or malignant nature [1]. Aneurysmal bone cysts were first described in 1942 by Jaffe and Lichtenstein [7], with only 2.7% involving the ribs [4]. The etiology of this lesion remains unclear, although it is hypothesized that they result from increased venous pressure, trauma, or pre-existing bone tumors, which eventually lead to bone resorption and the formation of a blood-filled cyst [4, 7–9].

These tumors usually occur in young individuals, with 75% of cases presenting before the age of 20 [9]. Although initial evaluations include CXR, CT plays a critical role in establishing the tumor location, composition, and involvement of adjacent tissues, serving as a guide for treatment planning [2]. Nonetheless, definitive diagnosis is almost exclusively histopathological, based on biopsy samples obtained via incisional, excisional methods, or wide resections [1].

In cases of suspected malignancy or intraoperative findings of positive margins, resection should be extended to achieve tumor-free margins of a minimum of 4 cm both proximally and distally, including adjacent rib segments, muscle, and underlying pleura. Reconstruction of the chest wall using osteosynthesis materials or mesh is recommended if more than four ribs are resected. For benign lesions or those with negative margins and low suspicion of malignancy, a margin of 1–2 cm is sufficient [2].

Additional measures, such as selective arterial embolization, sclerotherapy, chemotherapy, and radiotherapy [10], are primarily considered neoadjuvant options to reduce lesion size and minimize hemorrhage risk. However, these methods are less effective due to the high risk of recurrence and the potential progression to sarcoma associated with radiotherapy [11].

In this case, the minimally invasive RATS approach provided excellent exposure, vascular control, and precise tumor resection. Postoperative outcomes were favorable, with a short duration of chest tube placement and hospital stay. This approach allows for careful and less invasive chest wall reconstruction, optimizing both therapeutic and aesthetic outcomes compared to conventional thoracotomy [4, 8, 10].

Given the potential for recurrence, with rates up to 20% [10, 12], regular follow-up is crucial. Monitoring with periodic radiographic imaging is essential to ensure clear surgical margins. Follow-up for 2–5 years is recommended by some authors [10, 13, 14].

## Conclusion

Aneurysmal bone cysts are a rare entity characterized by rapid growth, potential involvement of surrounding tissues, and a high risk of hemorrhage. While traditionally managed via thoracotomy, RATS offers a significant advantage by providing enhanced visualization of the tumor’s full dimensions and enabling precise, en bloc resection with clear margins and superior aesthetic outcomes. Depending on the size of the lesion, these tumors can be effectively treated using minimally invasive techniques.
